# A Systematic Review on Suitability of Molecular Techniques for Diagnosis and Research into Infectious Diseases of Concern in Resource-Limited Settings

**DOI:** 10.3390/cimb44100300

**Published:** 2022-09-21

**Authors:** Akua K. Yalley, Selasie Ahiatrogah, Anna A. Kafintu-Kwashie, Gloria Amegatcher, Diana Prah, Akua K. Botwe, Mildred A. Adusei-Poku, Evangeline Obodai, Nicholas I. Nii-Trebi

**Affiliations:** 1 Department of Medical Laboratory Sciences, School of Biomedical and Allied Health Sciences, University of Ghana, Accra P.O. Box KB 143, Ghana; 2 Department of Obstetrics and Gynaecology, College of Medicine, Pan African University of Life and Earth Sciences Institute, University of Ibadan, Ibadan P.O. Box 22133, Nigeria; 3 Department of Medical Microbiology, University of Ghana Medical School, Accra GA-221-1570, Ghana; 4 West African Centre for Cell Biology of Infectious Pathogens, University of Ghana, Accra P.O. Box LG 54, Ghana; 5 Molecular Biology Unit, Kintampo Health Research Centre, Ghana Health Service, Kintampo P.O. Box 200, Ghana; 6 Department of Virology, Noguchi Memorial Institute for Medical Research, University of Ghana, Accra P.O. Box LG 581, Ghana

**Keywords:** molecular diagnostics, polymerase chain reaction, tropical diseases, infectious diseases

## Abstract

Infectious diseases significantly impact the health status of developing countries. Historically, infectious diseases of the tropics especially have received insufficient attention in worldwide public health initiatives, resulting in poor preventive and treatment options. Many molecular tests for human infections have been established since the 1980s, when polymerase chain reaction (PCR) testing was introduced. In spite of the substantial innovative advancements in PCR technology, which currently has found wide application in most viral pathogens of global concern, the development and application of molecular diagnostics, particularly in resource-limited settings, poses potential constraints. This review accessed data from sources including *PubMed*, *Google Scholar*, the *Web of Knowledge*, as well as reports from the World Health Organization’s Annual Meeting on infectious diseases and examined these for current molecular approaches used to identify, monitor, or investigate some neglected tropical infectious diseases. This review noted some growth efforts in the development of molecular techniques for diagnosis of pathogens that appear to be common in resource limited settings and identified gaps in the availability and applicability of most of these molecular diagnostics, which need to be addressed if the One Health goal is to be achieved.

## 1. Introduction

All over the world, and especially in African countries, infectious diseases constitute a major public health challenge and thus represent one of the greatest potential barriers to achieving the third Sustainable Development Goal. This is because, collectively, they account for approximately 20% of mortality in all age groups. In the least-developed countries, they contribute to about 33% of mortality (WHO, Geneva, Switzerland, 2006).

The infectious disease burden remains alarming the world over. Approximately 15 million people die each year because of tropical infectious diseases, with most of them living in developing countries [1]. The significance of neglected tropical diseases, most of which are poverty-driven, can be underscored in this: in May 2013, the 66th World Health Assembly of the WHO adopted a resolution, WHA66.12, requiring member states to pursue and intensify measures aimed at improving the health and social well-being of affected populations [2]. Considerably, infectious diseases such as HIV/AIDS, tuberculosis, and malaria have received significant global attention, and many have already been well-documented with appreciable references [3,4,5]. However, the same cannot be said of most neglected tropical infectious diseases. It is worthy of note that tropical diseases are not limited to the tropics. Globalization and accompanying increase in international air travel for purposes including migration, tourism, and work visits to tropical regions [6] have contributed to an equally increased incidence of tropical diseases in areas such as the United States, United Kingdom, and Europe. Surveillance and measures of effective control of infectious disease pathogens therefore represent important approaches for dealing with the global spread and threat of tropical and infectious diseases [7].

Various traditional methods exist for diagnosis of most infectious disease pathogens. Table 1 presents a cross-section of these methods. However, factors that affect the concentration of pathogens in blood or blood fractions, such as latency infections, tend to render plasma concentration of pathogens such as Ebola virus, malaria parasite, human immunodeficiency virus (HIV), and tuberculosis too low to be definitively determined by methods like ELISA or blood smear. Highly sensitive techniques are therefore required that are cost effective, have fast turn-around time, and also assure reliable detection of pathogens [8]. Invariably, almost every pathogen has a nucleic acid component, which makes it possible for molecular methods to be applied for their diagnosis, monitoring, and disease study. A few examples of the traditional molecular methods include conventional PCR, real-time PCR, chromatin immunoprecipitation analysis (ChIP), nested PCR, and multiplex PCR (real-time or conventional) [9,10,11,12]. In view of their time-tested sensitivity and specificity, molecular methods offer a very reliable means of infectious disease diagnosis. The growing challenge of the tropical and infectious disease burden makes advances in molecular methods as the mainstay of infectious disease pathogen detection and control imperative.

The need for molecular diagnostics that advance clinical care and public health delivery has never been greater. Nevertheless, there are untapped opportunities that can be harnessed in shaping technologies to address current unmet needs [13]. Emerging technologies are therefore warranted that enable the detection and quantification of pathogen burden with agility, sensitivity, and simplicity. It must be acknowledged, however, that significant challenges remain with regards to the development, regulatory approval, and integration of new technologies for use in clinical diagnostics. Considerable hurdles with using some molecular methods include the fact that they are relatively expensive; require cumbersome instrumentation and reliable electricity, among others; and often require a high level of technical expertise, thus constituting a disadvantage. This underscores the need for developing point-of-care (POC) molecular diagnostic methods that may overcome some of the challenges surrounding use of traditional molecular methods.

**Table 1:** Traditional or non-nucleic-acid-based methods of infectious disease diagnosis.

Infectious Disease	Method	Description (Common Procedures)	Challenges	Reference
Yaws	Microscopic examination	This method is used for the diagnosis of yaws at stage 1 and 2 using tissue samples from skin lesions.	Sensitivity is low when bacterial load is low, or treponemes viability is poor.	[14]
	Serological testing	Tests include rapid plasma reagin (RPR) and *Treponema pallidum* particle agglutination (TPPA).	Methods unable to distinguish yaws from syphilis.	[14]
Buruli ulcer	Microscopic examination	Involves direct smear or biopsy examination to detect acid-fast bacilli.	Low sensitivity.	[15]
	Cell culture of *Mycobacterium ulcerans* (MU)	Cell culturing to isolate viable MU for typically 9 to 16 weeks at 29–33 °C is a confirmatory test.	Culturing can take months.	[16]
	Histopathology	Analysis is done on tissue specimens in formalin stained with eosin and hematoxylin or other stains.	Method is expensive and does not always provide clear-cut identification.	[17]
Human African trypanosomiasis	Serologic testing	Used for screening purposes only.	Reliable test available only for *T.b. gambiens*.	[11]
	Microscopic examination	Used for the staging of both *T.b. gambiense* and *T.b. rhodesiense* using CSF.	Very low sensitivity.	[11]
Ebola	Cell culture	Confirms presence of Ebola virus. Visualization is done either directly by electron microscopy or indirectly by immunofluorescence microscopy.	Biosafety level 4 containment is required.	[18]
	Antibody detection	Detects antibodies in serum (of some healthy individuals) usually after 3 weeks.	Time taken for antibody to be detected after infection is too long.	[19,20]
Onchocerciasis	Microscopic examination	A gold standard. This is based on the detection of microfilariae in skin snips.	Sensitivity of the skin snip diminishes with decreasing skin microfilaria density.	[21]
	Slit-lamp examination	Procedure involves examination of the cornea and anterior chamber of the eye.	Onchocerciasis is not the only illness that may cause ocular lesions. Lesions may be seen in other infections also.	[22]
	Serological testing	The gold standard for diagnosing most common *Wuchereria bancrofti* cases is antigen detection. Antibody testing also exists.	Has extensive antigenic cross-reactivity with other nematodes. Antibody test is unable to distinguish current from past infection.	[23]
	Diethylcarbamazine (DEC) Patch Test	Papule formation after application of DEC to skin confirms the presence of microfilariae.	Issues with sensitivity decreases after treatment with ivermectin.	[24]

Indeed, there are emerging modern methods, such as HDA, NASBA, RPA, and LAMP, which are all isothermal technologies that have the advantage of not needing cumbersome equipment and are generally good alternatives to use in resource limited settings [25]; however, the realization of this need in developing countries is in itself another challenge. Of note, despite the increasing availability and complexity of diagnostic tests in developed countries, in the case of developing countries, though they bear the majority of the infectious diseases burden, they do not have adequate access to advanced diagnostic methods but largely depend either on clinical diagnosis or rapid point-of-care testing [26,27]. This systematic review therefore focuses on the utility of some molecular methods for diagnosing, monitoring, and studying infectious diseases often associated with tropical settings, with particular reference to some selected neglected infectious diseases, namely yaws, Buruli ulcer, sleeping sickness, Ebola, and onchocerciasis for illustration.

## 2. Methods

**Figure 1:**
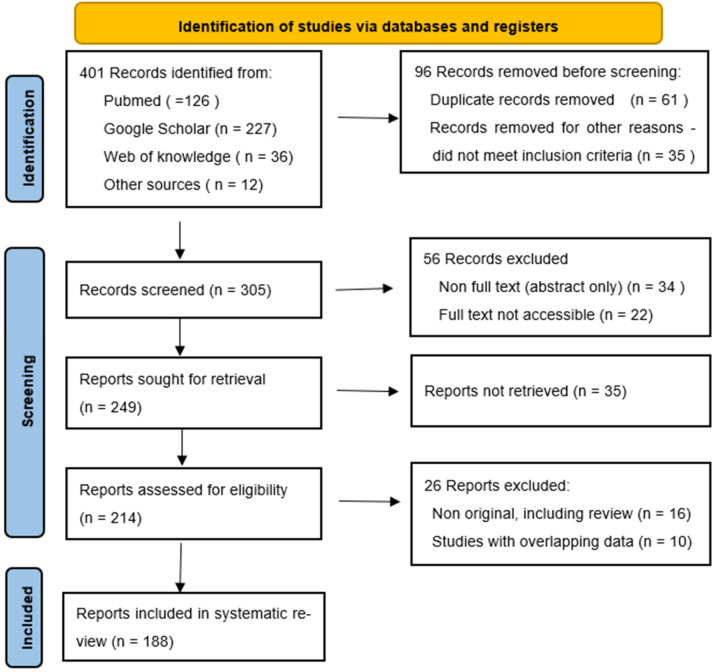
PRISMA 2020 flow diagram showing the search strategy, the number of records identified, and the number of included/excluded records.

The present systematic review follows the Preferred Reporting Items for Systematic Reviews and Meta-Analyses (PRISMA) guidelines. The sources of information used in this review were mainly peer-reviewed articles retrieved from *PubMed*, *Google Scholar*, and *Web of Knowledge* searches as well as reports from the World Health Organization’s Annual Meeting on infectious diseases. The search was limited to studies and reports published between 1 January 1965 and 30 June 2022. An advanced search tool together with a variety of themes connected to molecular techniques for infectious disease diagnosis were used in the search. The strategy employed is as illustrated in Figure 1 [28]. The keywords used include “resource-limited countries”, “molecular techniques”, “infectious disease”, “Yaws”, “Buruli ulcer”, “Ebola disease”, “Trypanosomiasis”, “Onchocerciasis”, and “challenges”. These keywords were put together using OR and AND Boolean operators where necessary. To optimize the quality of the information retrieved, the articles obtained were filtered using the title, abstract, or full text. Publications concerning molecular techniques used for diagnosis of infectious diseases were considered as the inclusion criteria; and articles that did not satisfy these criteria were excluded. Articles on molecular techniques that are unrelated to infectious disease diagnosis were not considered. All eligible articles/reports were imported to EndNote software X9 (Thompson and Reuters, Philadelphia, PA, USA), and duplicates were removed before further assessment.

## 3. Results and Discussion

The types of article retrieved included research, reviews, and abstracts. The goal was to gather as many materials as possible. However, unrelated materials were excluded.

### 3.1. Molecular Techniques as Applied to Yaws

The bacterium *Treponema pallidum* subsp. *pertenue* (TPE) causes yaws, a severe childhood infectious disease [14]. Yaws is now known to be prevalent in 13 nations. Some of those nations include Papua New Guinea, Solomon Islands, and Ghana [29]. However, adequate reporting data are limited [30,31]. Ghana, Papua New Guinea, and the Solomon Islands in the Southwest Pacific Ocean have reported the most incidences worldwide [29]. Yaws is spread through skin-to-skin contact [32]. Spirochetal bacteria, such as the Treponema species, are responsible for a group of diseases referred to as treponematoses. These diseases and the specific causative species are: yaws, caused by *Treponema pallidum pertenue*; pinta, caused by *Treponema carateum*; bejel, caused by *Treponema pallidum endemicum;* and the venereal disease syphilis, caused by *Treponema pallidum* [33].

Conversely, with respect to diagnosing yaws, healthcare personnel in yaws-endemic nations have two fundamental problems. First, TPE, which causes yaws, shares about 99.8% of its genomic structure with *T. pallidum* subsp. *pallidum* (TPA), the causative bacterium of syphilis [34]. As a result, all of the existing serological diagnostic techniques that detect yaws also detect syphilis [35]. Over the last two decades, there has been a growing drive using PCR techniques for treponematosis investigation. Detecting treponemal DNA using PCR only needs few treponemal chromosomal copies. As an in-house test, a number of sequences have been targeted. They include tpf-1, bmp, tpp47, tmp A, and pol A, among others [36,37,38,39,40]. These tests could be used to detect treponemes from swab specimens though they are not subspecies-specific. Unlike the case in swab samples, PCR’s utility in blood samples is generally hampered by the low amount of treponemes found in blood [41,42,43].

In recent times, methods for differentiating *T. pallidum* subspecies have been developed. These include a real-time PCR assay, nested PCR, a combination of PCR/RFLP analysis, and sequencing analysis [44,45,46,47,48,49,50]. Since some yaws variants harbor a primer binding-site mutation, which tends to yield false-negative PCR results, molecular diagnosis in such a case should employ primers that are targeted towards highly conserved regions [47]. An emerging feature regarding proper sample handling and storage for a successful PCR reaction involves utilization of dry swabs transported at room temperature, which has been demonstrated to perform just as well in PCR as compared to swab stored in a carrier medium and subsequently transported using a cold chain [51]. This holds promise that in resource-limited settings, the use of dry swabs to cut down costs will not compromise PCR outcomes.

Despite their utility and reliability, molecular technologies such as PCR are not readily available in the field [52], in which case other techniques such as isothermal nucleic acid amplification has some relevance, as they have some advantages compared to traditional PCR. Furthermore, unlike PCR, a technique such as loop-mediated isothermal amplification (LAMP) does not necessitate thermal cycling, thereby removing the challenges associated with using a thermal cycler [53]. LAMP therefore is an ideal technique for use in developing point-of-care (POC) tests [54,55]. Indeed, a LAMP assay that can deferentially diagnose *T. pallidum* and *H. ducreyi* (targeting (pol A) gene and 16S rRNA, respectively) has recently been developed (TPHD-LAMP) with an impressive diagnostic performance of 85–92% and 85–96% sensitivity and specificity, respectively [55]. A higher sensitivity of 100% and 96% specificity of the LAMP assay (compared to a CDC real-time PCR assay) targeting the tp0967gene of *T. pallidum* has also been reported [35].

A TPHD-RPA assay, which is based on the RPA technology, was developed by Frimpong et al. [56] to simultaneously and rapidly detect *H. ducreyi* and *T. pallidum*. The genes targeted were pol A for yaws and the hemolytic cytotoxin HhdA gene for *H. ducreyi.* The assay was demonstrated to have 94–95% and 100% sensitivity and specificity, respectively [56] (Frimpong et al., 2020). RPA technology appears to have some advantage over LAMP in that it has a shorter turn-around time (15 min as compared to 30 to 60 min in the case of LAMP) at 37 to 42 degrees [57,58]. To some extent, the technology has been successfully utilized outside typical lab settings in low-resource environments [59,60,61].

### 3.2. Molecular Techniques Applicable for the Diagnosis of Buruli Ulcer

Buruli ulcer disease (BUD), caused by *Mycobacterium ulcerans* (MU), is a skin infection that results in leg and arm ulcers and, if left untreated, can permanently disfigure affected individuals. The disease is generally found in the tropics as well as subtropics, such as west Africa and Asia [62,63]. Transmission is more frequent amongst people residing close to water bodies [64]. Currently, the main laboratory methods used in diagnosing/investigating BUD are culture, microscopy, histopathology, and nucleic acid detection methods such as PCR [15,16,17,65,66]. To confirm BUD diagnosis, the WHO recommends two laboratory tests or one positive microscopy/PCR test in endemic areas [67] (WHO, 2008a; WHO, 2008b).

Despite existence of various diagnostic methods for BUD, PCR is the accepted gold standard. Samples that can be used for PCR include swabs, fine-needle aspirates, and tissue specimens [68,69,70]. The PCR technique specifically targets the sequence referred to as IS2404, giving it its high sensitivity and specificity [71]. Accordingly, the WHO indicates that a positive PCR test result is regarded as enough evidence to start an anti-mycobacterial regimen [67] (WHO, 2008a). It might, however, not be the best tool (compared to culture) to monitor treatment success, as it has been found that the presence of MU DNA persists long after lesions have been treated [72].

Conventional PCR, nested PCR, and real-time PCR have been used for BUD investigation, targeting a number of sequences, including IS2404, IS2606, hsp65, rpoB gene, and 16srRNA gene [64,65,73,74,75,76,77,78,79,80]. Some of the genes targeted are genus- rather than species-specific, and therefore, this necessitates the need to combine with other methods such as restriction fragment length polymorphism (RFLP), sequencing, and oligospecific capture plate hybridization for species differentiation [78,79,80]. Nevertheless, PCR targeting IS2404 has been shown to be more specific, with real-time PCR being more sensitive as compared to conventional PCR [64,70,74,75,81]. Moreover, real-time PCR reduces the possibility of contamination with amplicons from previous reactions. Other techniques employed for BU investigation include LAMP assays (targeting the IS2404 sequence among others) [71,82,83,84], with sensitivities comparable to general conventional PCR but not to real-time PCR [83,84]. A major challenge surrounding the use of LAMP is its adaptability for use in field settings with respect to generating isothermal conditions as well as performing nucleic acid extraction and purification. Thus, the development of DRB-LAMP that utilizes lyophilized reagents and with sensitivity comparable to that of conventional LAMP assay is a laudable approach [85]

A recently developed assay, the RPA for BU diagnosis (targeting the IS2404 sequence) [86], which has short turn-around time. operates at lower isothermal temperatures (compared to LAMP), and has appreciably high specificity and sensitivity of 100% and 88%, respectively, compared to real-time PCR, represents a significant achievement with regards to BU molecular diagnosis.

### 3.3. Molecular Techniques Applicable for the Diagnosis of Human African Trypanosomiasis (HAT)/Sleeping Sickness

Sleeping sickness is a parasitic illness mainly spread by tsetse flies. It is caused by two protozoan parasites from the Trypanosoma genus, resulting in two forms of the disease—*Trypanosoma brucei gambiense* and *Trypanosoma brucei rhodesiense* [87]. *Trypanosoma brucei* (T.b.) *gambiense* is mainly found in Western and Central Africa, accounting for a majority of cases and can be prolonged for months or years [88]. *Trypanosoma brucei* (T.b.) *rhodesiense* is mainly found in Eastern and Southern Africa, where it accounts for a minority of cases, causes acute infections, and is considered zoonotic [88,89,90]. Clinical features of the disease can mimic that of other diseases such as malaria. As such, laboratory testing of any suspected case is imperative [91]. In general, diagnoses of HAT is divided into three steps, namely screening, followed by confirmation, and then staging [87]. For screening and confirmation, an antibody-based card agglutination test (CATT/*T. b. gambiense*), microscopy, as well as RDTs have been used [11,92,93]. However, issues with sensitivities and specificities necessitate the need to also include molecular methods among assays used to diagnose the disease, and a number of these that either detect DNA or RNA have been developed.

Conventionally, nested and real-time PCR assays have been employed that target sequences such as ITS1 DNA and ESAG6/7 gene satellite DNA, among others [94,95,96]. These targets are generally not sub-species-specific. A few sub-species PCR assays in use target sequences such as the TgsGP and SRA gene [97,98,99,100,101,102]. However, these specific assays are generally less sensitive in that the target sequences have relatively fewer copy numbers [97,98,99]. Other assays for subspecies differentiation targeting the SRA and TgsGP sequences have also been used and shown to be more sensitive than their PCR counterparts, which is very encouraging [103,104,105,106].

### 3.4. Molecular Techniques Applicable to Ebola Disease Diagnosis

Ebola virus disease (EVD) is caused by a virus that belongs to the Filoviridae family of viruses. There are currently six species belonging to the Ebolavirus genus, namely Soudan ebolavirus (SUDV), Zaire ebolavirus (ZEBOV), Bundibugyo ebolavirus (BDBV), Tai Forest ebolavirus (TAFV), Bombali ebolavirus (BOMV), and Reston ebolavirus. [107,108,109]. BDBV, TAFV, SUDV, and ZEBOV are all known to infect humans [107,108,109]. Ebolavirus has been responsible for at least 20 disease outbreaks, with devastating consequences [110]. Fatality rates have ranged from 39.5% to 100%, and early symptoms mimic that of a variety of less severe diseases; thus, the need to diagnose and isolate as early as possible cannot be over emphasized [110].

Diagnostic methods used include antigen tests, electron microscopy, cell culture, antibody detection tests, and nucleic-acid-based tests. They each have their challenges. For instance, with the antibody tests, antibodies are detected in some “healthy individuals”, and as is common with serological tests, it takes about 3 weeks after infection before antibodies can be detected [19,20]. Antigen tests have a number of sensitivity and specificity issues [111]. Currently, real-time RT-PCR is the gold standard used for EVD diagnosis due to its sensitivity [111,112,113]. Several bodily fluids can be used for PCR detection, and they include blood, urine, saliva, sweat, vaginal fluid, and semen, among others [110,113,114,115]. PCR-based assays often target the glycoprotein and/or the nucleoprotein; usually, the two gene target assays do not need to be repeated for confirmation [116,117]. An assay such as RealStar^®^ Zaire Ebolavirus RT-PCR 1.0 specifically targets the species ZEBOV. Others such as “RealStar^®^ Filovirus Type RT-PCR 1.0” and the “RealStar^®^ Filovirus Screen RT-PCR 1.0” can distinguish between the Marbug virus genus, which also belongs to Filoviridae family of viruses, and five ebolavirus species [116,118].

In addition to the normal challenges, such as longer turn-around time for results and requiring highly skilled personnel, which are often associated with traditional PCR, Ebola virus is a very deadly pathogen, so very stringent containment procedures such as a BSL-4 hood or a portable equivalent as well as efficient inactivation of the virus prior to testing are required. In order to address some of these challenges, automated assays such as Cepheid’s GeneXpert Ebola assay and Biofire’s Film Array Ebola assays have been developed [119,120,121]. GeneXpert is a fully automated system that has the ability to inactivate the virus and perform extractions, amplifications, and detection in less than 2 h. The assay targets two genes, namely the glycoprotein and nucleoprotein [119,120,122]. Specificity and sensitivity of the assay is very high, reaching up to 100% sensitivity and 99.5% specificity, according to some reports, compared to a traditional real-time PCR assay [119,122,123]. The Film Array Ebola assay is also another automated system that uses a nested multiplex coupled with melt curves to determine results within an hour [121,124]. Sensitivity and specificity of the assay ranges from 75–84% and 89–100%, respectively, according to one study [121]. This makes it another alternative when considering the use of a potential point of care device in a resource-limited setting.

Other PCR-based devices/assays developed that aim to address challenges with the traditional PCR systems for Ebola include the on-chip RT-PCR system that can detect virus in as little as 7.5 min using regular sample volume. In addition to this is the palm-sized on-chip device, which is capable of detecting virus in less than 40 min and whose size would be very conducive for point-of-care testing [125,126]. RT-LAMP assays targeting the nucleoprotein gene or the glycoprotein gene have been developed with variations [127,128,129,130]. For example, not all RT-LAMP assays require samples to be extracted prior to use [127]. The extraction-free type, which also uses lyophilized reagents, makes it easier for it to be used as a point-of-care assay [127]. Further, Ebola RPA and RT-RPA assays have recently been developed with limit of detection and sensitivities comparable to that of traditional real-time PCR [131,132]. The current draw back for both is having to perform the RNA extraction separately [131,132].

Of note, sequencing plays a major role in confirming the introduction of variants in a country as well as confirming reinfection among people. Fortunately, bench-top sequencers and portable bench-top sequencers have all been used in endemic regions to identify variants [133,134,135,136,137]. One example is the use of the MinION (Oxford Nanopore Technologies) portable nanopore sequencing technology, which shortens the time it takes to obtain the genome sequence from subject specimens by half, and in the 2014/2015 West African outbreaks, this sequencing technology allowed EBOV reinfection among people in Guinea and Sierra Leone to be confirmed promptly [135].

### 3.5. Molecular Techniques Applicable for the Diagnosis of Onchocerciasis/River Blindness

The filarial parasite *Onchocerca volvulus* causes onchocerciasis, also known as river blindness. The parasite has infected 37 million individuals, mainly in the Sub-Saharan region of Africa, and individuals who are estimated to be in danger of contracting onchocerciasis are over 100 million [138,139]. The WHO classifies onchocerciasis as a neglected tropical disease (NTD). Monitoring the levels of *Onchocerca volvulus* transmission is critical for assessing the efficiency of national onchocerciasis control programs. Customarily, the identification of larvae by dissection of flies and subsequent microscopy has been used to estimate the transmission potential of *Simulium* vector populations. With regards to diagnosis, examining skin snips under the microscope is the traditional technique. However, this method is not viable for routine surveillance of the vector since it is exceedingly labor-intensive [21]. More importantly, it is an insensitive method for diagnosis [140].

Other tests for the diagnosis of onchocerciasis include the diethylcarbamazine (DEC) patch and antibody tests [23,24]. Drawbacks to those tests include questionable specificity for the patch test and inability to distinguish between current and past infections for the antibody test [23,141,142]. Hence, PCR and PCR-based methods have been developed for diagnosis and entomological surveillance. The first PCR-based assay targeted a 150-base-pair repeat in the *O. volvulus* genome (O-150) [143]. The amplification was coupled with hybridization using a probe that is species-specific [143,144]. Furthermore, sensitive real-time PCR assays have been developed that target the O-150, COX-I locus, rDNA genes, and O-5S rRNA gene [140,145,146,147]. Of these, the O-5S real-time PCR assay has been shown to be more sensitive than both microscopy and the O-150 real-time PCR [147]. What remains a bit of a challenge is sequencing and stem-loop RT-qPCR assays for *Onchocerca volvulus* parasitic miRNA detection, which, though they are in existence, are currently not widely used diagnostic methods due to sensitivity issues owing to low levels of detectable miRNA [148,149].

LAMP assays have been successfully developed targeting cox1 and glutathione S-transferase 1a (OvGST1a) genes [150,151,152,153]. The LAMP assays have been shown to exhibit high levels of specificity and sensitivity compared to PCR. It is important, though, to note that targeting the cox1 gene in the assay encounters cross-reactivity with *Onchocerca ochengi*, which typically infects cattle but not humans [150,154], thus constituting the assay’s main drawback. Nevertheless, the development of LAMP assays for surveillance as well as diagnostics is a step in the right direction due to the advantage of ease-of-use for point-of-care testing that they have over traditional PCR and other methods. Table 2 highlights the molecular techniques described for the diagnosis of yaws, Buruli ulcer, HAT, Ebola, and onchocerciasis.

## 4. Discussion

This systematic review of the utility of molecular methods for diagnosing, monitoring, and studying neglected infectious diseases common to the tropics has focused on yaws, Buruli ulcer, sleeping sickness, Ebola, and onchocerciasis and serves as a useful resource for research and patient care in endemic areas.

### 4.1. Comparative Usefulness of Molecular Techniques for Infectious Disease Diagnosis

Typical turn-around time for LAMP assays is short as compared to conventional PCR. Specifically, it takes about 30 min to one hour at 60 to 65 °C [56,161]. Furthermore, the method uses more robust reagents that can easily be transported under suboptimal conditions [161]. However, one disadvantage associated with LAMP assays generation is that it requires the design of four to six primes per assay, which can be challenging, even though that increases the specificity of the assay [56,162]. Invariably, these examples demonstrate that the development of effective isothermal (NAAT) assays is a step in the right direction due to their relative ease of use in low-resource settings for differential disease diagnoses, monitoring drug resistance, as well as general disease screening. It is important, however, to mention that, among the challenges faced by some resource-limited settings, there is a need for adequate facilities, such as fridges and freezers to store reagents and samples. It is, therefore, in order to help address such challenges that lyophilized reagents have been developed and successfully used in PCR (DRB-PCR) to detect MU [163,164], for example. The convenience of use of the RPA assay coupled with its relatively high sensitivity and specificity makes it a promising assay not only for point of care tests but also field testing and testing in resource-limited settings.

### 4.2. Gaps

There is need for point-of-care tests and expansion in usage of nucleic-acid-based molecular technologies in resource-limited settings. Some of the newer technologies such as the RPA technology, developed by Frimpong et al. [56] and shown to have some advantage over LAMP in having a shorter turn-around time (15 min vs. 30 to 60 min for LAMP) at 37 to 42 degrees [57,58], have had very limited successful usage outside a typical laboratory setting in low-resource environments [59,60,61]. Furthermore, the need to create isothermal conditions and also to conveniently perform nucleic acid extraction and purification in a field setting represent some of the major challenges of LAMP if it has to be applied in the field setting, as the assay sensitivity is reduced drastically when crude extracts are used [71,84]. These challenges remain to be surmounted.

Another important issue to consider in making molecular techniques widely available and user-friendly in resource-limited settings is that of cost. Serious efforts have to be put in to make the techniques accessible and affordable in almost every research and diagnostics establishment. Invariably, a molecular technique such as fluorescent in situ hybridization (FISH) has been used to detect trypanosomes but might be a challenge to use in resource-limited settings due to the cost involved, among other things [158,159].

### 4.3. The Way Forward

Although molecular assays are a good choice in that they are more sensitive than the non-molecular methods, one concern that warrants attention in the diagnosis of certain infection conditions is that DNA may remain detectable in treated patients as well as those with latent infection [165,166,167]. This is noteworthy for clinicians when interpreting results from DNA-based assays, and where possible, they might consider opting for RNA-based assays since detecting RNA in a patient implies an active infection [168,169]. For example, in the case of human African trypanosomiasis, RNA assays in use include reverse transcriptase real-time PCR and NASBA [155,169,170,171], which target the SL RNA or 18SrRNA for pathogen detection. This underscores the need for further developments in molecular technologies that offer alternatives for definitive detection of various pathogens in various circumstances. In that pursuit, however, the fact that RNA is more prone to degradation from nuclease action than DNA may represent a setback or challenge, as is the case with NASBA, and therefore would require critical approaches such as choice of reagents and use of sterile techniques for successful and reliable assay outcomes.

The need to design equipment and assays that are robust and can maintain efficient functioning and durability even in adverse conditions such as unclean environments, as may pertain in field settings, is another factor. A typical example is the disadvantage associated with the GeneXpert, which has problems when used in dusty environments in several instances [172]. This represents a challenge for adapting molecular technologies for use in certain field settings.

## 5. Conclusions

Infectious diseases pose a significant public health risk, and infectious disease epidemics can have substantial social, political, and economic consequences. Past outbreak situations offer ways for designing effective response to infectious disease events. In such situations, molecular diagnostics have important application in screening and confirmation for asymptomatic infections, syndromic therapy, as well as prevention of long-term sequelae. As such, their relevance in disrupting disease transmission and disease eradication efforts cannot be overemphasized. However, sometimes even with increased sensitivity, a positive molecular test does not always indicate the presence of causal microbes; hence, it is important that results must be evaluated together with the clinical picture and other supplemental exams. In implementing molecular diagnostics, technical complexity and instrumentation difficulties represent unique hurdles in endemic regions and resource-constrained settings, but isothermal methods (for example, LAMP) promise more complete application in such settings. Fortunately, all the diseases considered demonstrated that isothermal methods of amplification are sufficiently developed for POC uses and could eventually lead to a reduction in the burden of infectious diseases in resource-limited settings.

## Figures and Tables

**Table 2:** Nucleic-acid-based methods of infectious disease diagnosis.

Infectious Disease	Method	Specimen Types Used	Sequence/Gene Targets	Challenges	Reference(s)
Yaws	PCR, (Conventional and real-time multiplex), TPHD-LAMP, TPHD-RPA, sequencing	Blood, ulcer sample (Swab)	tpf-1, bmp, tpp47, tmpA, pol A, tp0967, other	The low amount of treponemes in blood limits PCR diagnosis of ETs from blood samples; for conventional and real-time PCR, the process is time-consuming and requires expensive laboratory equipment.	[36,37,38,39,40,41,42,43,49,50,55,56]
Buruli ulcer	PCR (Conventional/real-time), RPA, LAMP	Swabs, fine-needle aspirates, tissue specimens	IS2404, IS2606, hsp65, rpoB, 16srRNA, 65kda-hsp and enoyl reductase genes, VNTR	The conventional PCR assay is less sensitive, more cumbersome, and time-consuming as compared to other molecular methods.	[64,65,73,74,75,76,77,78,79,80,83,86]
Human African trypanosomiasis	PCR, (conventional, nested, real-time), LAMP, RPA, NASBA, fluorescence in situ hybridization (FISH)	Blood and CSF	ITS1 DNA, ESAG6/7, satellite DNA, TgsGP, SRA, 18SrRNA and PFRA genes, RIME, and SL RNA	The main challenge with the RNA-based assays is that RNA is more susceptible to degradation compared to DNA.	[97,98,99,100,101,102,155,156,157,158,159]
Ebola	PCR (Conventional, real-time and real-time-based assays), LAMP, RPA, sequencing (Oxford nanopore)	Blood, urine, saliva, sweat, vaginal fluid, semen, other body tissue	Glycoprotein, nucleoprotein	The conventional PCR assay is less sensitive, cumbersome, and slow as compared to other molecular methods.	[116,117,127,131,132,135]
Onchocerciasis	Conventional PCR, real-time PCR, LAMP, stem-loop RT-qPCR, sequencing	Skin snips	O-150, cox1, glutathione S-transferase 1a (OvGST1a), O5-S, rDNA genes, O-5S rRNA gene, *Onchocerca volvulus* parasitic miRNA	The conventional PCR assay is less sensitive, cumbersome, and slow as compared to other molecular methods.	[140,143,145,146,147,148,149,150,151,152,153,160]

## Data Availability

Not applicable.

## Abbreviations

rpoB	Beta subunit of RNA polymerase
BDBV	Bundibugyo ebolavirus
BOMV	Bombali ebolavirus
BSL-4	Biosafety level 4
BUD	Buruli ulcer disease
CATT	Card agglutination test
ChIP	Chromatin immunoprecipitation
COX-I	Cyclooxygenase-1
CSF	Cerebrospinal fluid
DEC	Diethylcarbamazine
DNA	Deoxyribonucleic acid
DRB-LAMP	Dry-reagent-based loop-mediated isothermal amplification
ELISA	Enzyme-linked immunoassay
ESAG6/7	Expression-site-associated genes 6/7
EVD	Ebola virus disease
FISH	Fluorescent in situ hybridization
HAT	Human African trypanosomiasis
HIV	Human immunodeficiency virus
hsp65	Heat-shock protein 65
ITS1	Internal transcribed spacer 1
LAMP	Loop-mediated isothermal amplification
miRNA	Micro ribosomal ribonucleic acid
MU	*Mycobacterium ulcerans*
NAAT	Nucleic acid amplification test
NASBA	Nucleic acid sequence-based amplification
NTD	Neglected tropical disease
O-150	150-base-pair repeat in the *Onchocerca volvulus* genome
OvGST1a	*Onchocerca volvulus* glutathione S-transferase 1a
PCR	Polymerase chain reaction
POC	Point of care
pol A	DNA polymerase I
rDNA	Ribosomal deoxyribonucleic acid
RDTs	Rapid detection tests
RFLP	Restriction fragment length polymorphism
RPA	Recombinase polymerase amplification
RPR	Rapid plasma reagin
rRNA	Ribosomal ribonucleic acid
RT-PCR	Reverse transcription polymerase chain reaction
RT-qPCR	Reverse transcription quantitative polymerase chain reaction
RT-RPA	Reverse transcription recombinase polymerase amplification
SL RNA	Spliced leader ribonucleic acid
SRA	Serum resistance associated
SUDV	Soudan ebolavirus
T.b.	*Trypanosoma brucei*
TAFV	Tai Forest ebolavirus
TgsGP	*T. gambiense*-specific glycoprotein
TPA	*Treponema pallidum* subsp. *pallidum*
TPE	*Treponema pallidum* subsp. *pertenue*
TPHD-LAMP	*T. pallidum* and *H. ducreyi* loop-mediated isothermal amplification
TPHD-RPA	*T. pallidum* and *H. ducreyi* recombinase polymerase amplification
TPPA	*Treponema pallidum* particle agglutination
WHO	World Health Organization
ZEBOV	Zaire ebolavirus
